# Colossal magnetic phase transition asymmetry in mesoscale FeRh stripes

**DOI:** 10.1038/ncomms13113

**Published:** 2016-10-11

**Authors:** V. Uhlíř, J. A. Arregi, E. E. Fullerton

**Affiliations:** 1 Center for Memory and Recording Research, University of California, San Diego, La Jolla, California 92093-0401, USA; 2 CEITEC BUT, Brno University of Technology, Purkyňova 123, 612 00 Brno, Czech Republic; 3 CIC nanoGUNE, Tolosa Hiribidea 76, E-20018 Donostia–San Sebastián, Spain

## Abstract

Coupled order parameters in phase-transition materials can be controlled using various driving forces such as temperature, magnetic and electric field, strain, spin-polarized currents and optical pulses. Tuning the material properties to achieve efficient transitions would enable fast and low-power electronic devices. Here we show that the first-order metamagnetic phase transition in FeRh films becomes strongly asymmetric in mesoscale structures. In patterned FeRh stripes we observed pronounced supercooling and an avalanche-like abrupt transition from the ferromagnetic to the antiferromagnetic phase, while the reverse transition remains nearly continuous over a broad temperature range. Although modest asymmetry signatures have been found in FeRh films, the effect is dramatically enhanced at the mesoscale. The activation volume of the antiferromagnetic phase is more than two orders of magnitude larger than typical magnetic heterogeneities observed in films. The collective behaviour upon cooling results from the role of long-range ferromagnetic exchange correlations that become important at the mesoscale and should be a general property of first-order metamagnetic phase transitions.

## Abstract (web summary)

FeRh possesses a unique hysteretic metamagnetic phase transition between antiferromagnetic and ferromagnetic order close to room temperature. Here, the authors demonstrate a strong enhancement of the asymmetry of this transition in mesoscale stripes of FeRh.

## Graphical abstract

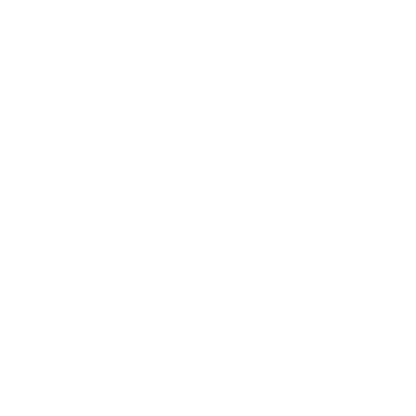

Understanding and ultimately controlling emergent phenomena at the mesoscale^1^ requires quantifying the interactions and correlations of the individual constituents in complex materials^2^,^3^,^4^,^5^, as well as engineered^6^,^7^ or self-assembled systems^8^. Interactions of strongly correlated electrons and symmetry breaking in materials often lead to ordered states such as charge ordering, superconductivity, ferromagnetism and multiferroicity featuring first-order phase transitions with coupled order parameters^9^. The nature of first-order phase transitions that exhibit an interplay between multiple degrees of freedom (that is, electronic, structural and/or magnetic) is at the forefront of materials science. Examples include metal-insulator transitions (MITs) in oxides (for example, manganites^10^ and VO_2_, ref. 11), the Verwey transition in Fe_3_O_4_ (ref. 12), and metamagnetic transitions from the antiferromagnetic order (AF) to ferromagnetic order (FM) in manganites^13^,^14^, CeFe_2_ alloys^15^, Mn_2_Sb alloys^16^, Hf_0.8_Ta_0.2_Fe_2_ (ref. 17) and FeRh^18^,^19^,^20^,^21^,^22^. In such systems, the nature of the transition can readily be tuned by external parameters such as strain^3^,^21^,^23^, pressure^24^,^25^, temperature, magnetic^21^,^26^ and electric fields^27^ and chemical doping^28^,^29^.

First-order phase transitions are generally characterized by hysteresis and phase separation at the transition^2^,^30^. This phase inhomogeneity coupled with local disorder often results in a broad transition as local regions undergo the transitions at different temperatures or fields^31^. One approach to studying the nature of the phase transition is to study materials at the scale of the inhomogeneity in the system, either using spatially sensitive probes or confining the material to the scale of the heterogeneity. For confined systems, the broad phase transition will often exhibit discrete jumps in the order parameter as discrete regions of the sample undergo the first-order transition^32^,^33^. For instance, the MIT in VO_*x*_ and manganites is exhibited by the temperature-dependent resistance changes occurring in a series of steps in patterned nanostructures, whereas continuous transitions are observed for films^34^,^35^. Transport measurements in a constricted geometry have further revealed considerable details about the nature of phase transitions and new phenomena such as the second re-emergent transition observed in nanostructured manganite films^36^.

Here we explore the heterogeneous nature of the metamagnetic phase transition in epitaxial FeRh films patterned into mesoscale stripes. FeRh undergoes a first-order phase transition from an AF to a FM phase (see Fig. 1a) upon heating from room temperature to above ∼360 K in zero magnetic field^18^,^21^. This first-order transition exhibits temperature hysteresis between heating and cooling cycles and the transition is accompanied by a volume increase of 1–2% (ref. 37), a reduction in resistivity^19^ and a large change in entropy^38^. The magnetoresistance and magnetization changes enable new metallic memory cells^39^, new approaches in magnetic recording^40^ and magnetic refrigeration^5^,^38^, and can be actively controlled with low power when coupled to a piezoelectric^23^. More fundamentally, this material has become a test-bed for exploring the interplay of structural, magnetic and electronic phase transitions in metallic systems including ultra-fast optically induced phase transitions^41^,^42^. In this study, we show that the metamagnetic phase transition becomes strongly asymmetric in mesoscale FeRh stripes. We argue that this results from the fundamentally different role of magnetic correlations in the AF and FM phases.

## Results

### Growth and characterization of FeRh films

**Figure 1:**
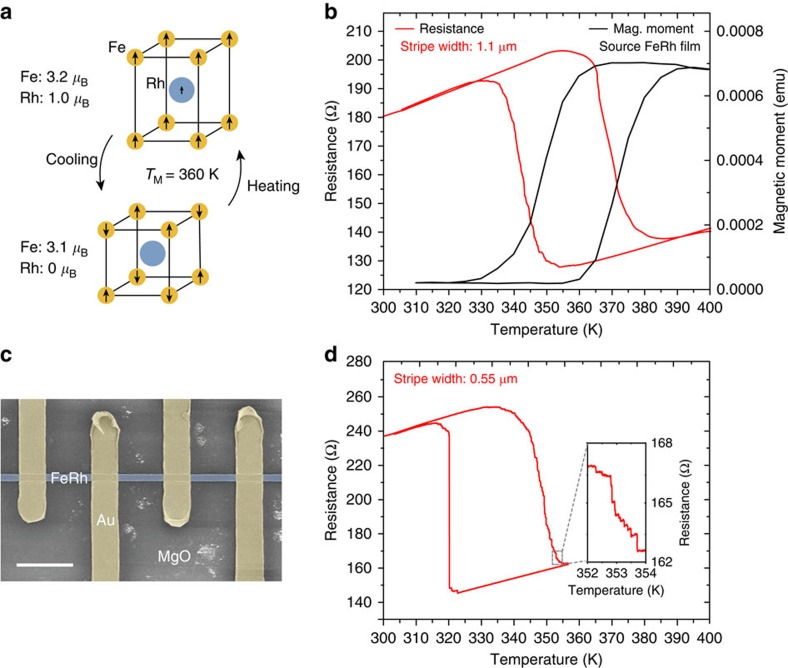
Magnetostructural and transport properties of FeRh thin films and patterned stripes. (**a**) The tetragonal cell of the AF FeRh film^21^,^22^ at room temperature expands its volume when heated through the transition temperature to the FM state. The orientation of the AF-coupled planes was revealed by neutron diffraction^22^. The magnetic moments per atom^22^ are indicated for each phase. (**b**) Net magnetic moment versus temperature of a 25-nm-thick FeRh film and resistance versus temperature of a 1.1-μm wide stripe patterned from the same film. (**c**) Scanning electron microscopy image of the patterned FeRh stripe with transport leads. Scale bar, 5-μm-long. (**d**) Asymmetry in the transition for a 33-nm-thick, 550-nm-wide and 4-μm-long stripe. Compared with full films and wide stripes, the phase transition is shifted to lower temperatures due to strain relaxation. The inset shows discrete steps in the order parameter upon heating corresponding to the transition in uncorrelated regions of the sample. Upon cooling, the transition proceeds primarily through a single event.

Figure 1b shows the temperature dependence of the net magnetic moment of a 25-nm-thick FeRh film grown epitaxially on an MgO (001) substrate (see Supplementary Figs 1–3 for structural details), together with the temperature dependence of the resistance of a 1.1-μm-wide stripe patterned from the same film. The onset of FM order with heating is seen as an increase of the magnetization, increase in the lattice constant (see Supplementary Figs 2 and 3) and concomitant decrease in the resistance. The resistance and magnetization across the phase transition are continuous smooth functions of temperature (or magnetic field^21^) yielding a transition that is relatively broad (about 10 K) as also seen in previous publications on FeRh films^21^. The broadening of the transition is linked to film heterogeneity originating from local structural variations where the first-order phase transition exhibits a phase coexistence of AF and FM domains with different transition temperatures as one progresses through the transition^23^,^31^,^43^,^44^,^45^. The coexistence of AF and FM domains has been imaged by a range of techniques revealing typical sub-micron AF and FM domain sizes^46^,^47^,^48^. The phase coexistence can also be inferred from temperature dependent X-ray diffraction (see Supplementary Figs 2 and 3 and ref. 49).

### Electrical transport measurement of FeRh stripes

We explore the nature of the phase transition by patterning the FeRh films into stripes (Fig. 1c) with lateral dimensions below 1 μm corresponding to the characteristic domain sizes typically seen in imaging^46^,^47^,^48^. We find that confining the lateral dimension allows us to track the phase transition in the individual domains via electrical transport experiments. When warming the sample from the AF to FM phase the width of the transition is similar to that observed for the film. However, since the domain sizes are comparable to the patterned sample size we observed a staircase-like transition (see inset of Fig. 1d for a 550-nm-wide stripe) as distinct regions undergo the metamagnetic transition. The resistance steps range from 0.1 to 3.0% of the total resistance change during the transition, corresponding to the FM activation areas of 0.002 to 0.08 μm^2^. The typical (median) step corresponds to regions of 0.005 μm^2^. Surprisingly, we observe a qualitative change in the transition behaviour upon cooling as seen in Fig. 1d. When cooling the FM-to-AF transition shifts to lower temperatures than observed in the film and occurs primarily in only a few discrete jumps. For the sample shown in Fig. 1d the majority of the sample switches at a single temperature (within a 0.01 K temperature change), indicating a collective response and almost a complete suppression of the phase separation across the entire sample even though the sample size (2.2 μm^2^) is much larger than the typical heterogeneous region observed in full film imaging.

**Figure 2:**
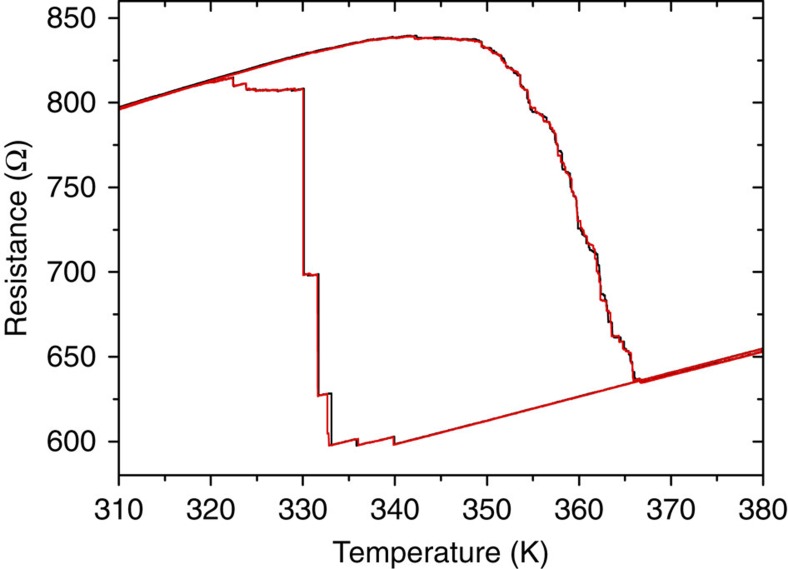
Reproducibility of the resistance scans. Dependence of resistance versus temperature for a 50-nm-thick, 220-nm-wide and 2.6-μm-long stripe. The transition upon heating is continuous and uncorrelated, whereas upon cooling the transition occurs in a few abrupt steps. Two hysteresis loops (black and red curves) measured in a sequence are plotted showing an excellent reproducibility upon temperature sweeping.

To understand the origins of the enhanced asymmetry in the phase transition in FeRh stripes we have explored the response of the patterned FeRh magnetic stripes on multiple films, different substrates and various measurement protocols. The asymmetry shown in Fig. 1d is a general observation for stripes of a width on the order of 500 nm or narrower. However, the exact behaviour can vary from sample to sample. Figure 2 shows resistance versus temperature for a 50-nm-thick, 220-nm-wide and 2.6-μm-long stripe where the cooling transition occurs in a few steps instead of only one isolated transition as seen in Fig. 1d. However, the sizes of the FM-to-AF jumps upon cooling are much larger than those seen in the warming curve. The thermal cycling was repeated (Fig. 2) and demonstrates high reproducibility for both the warming and cooling curves. This suggests that the number and size of steps upon cooling is linked to the local microstructure and is not governed by random thermally-activated processes. Although the number of discrete jumps varies even for the stripes of identical size fabricated from the same film, the asymmetry in the properties with heating and cooling is consistently present. Therefore, the presence of structural heterogeneity does not, on its own, explain the qualitative difference between the AF-to-FM and FM-to-AF transitions.

We have further explored the role of the film structure on the asymmetric transition by comparing the results for FeRh films grown on MgO (001) and Al_2_O_3_ (0001) substrates^21^. Details of the typical film structure are shown in Supplementary Figs 1–3 and discussed in ref. 21 for similarly grown films. These two substrates provide a different strain state of the FeRh film (Al_2_O_3_ induces tensile in-plane strain while MgO induces compressive strain). This strain shifts the transitions to higher temperatures on MgO and to lower temperatures on Al_2_O_3_. Furthermore, MgO (001) substrates yield FeRh (001) films while Al_2_O_3_ (0001) yield FeRh (111) that is twinned on the Al_2_O_3_ substrate. Despite this significant structural difference we observed a similar asymmetry in the transition when patterned into stripes (Supplementary Fig. 4) demonstrating that the asymmetry is not the result of strain relaxation due to patterning^50^ or specifics of the crystallographic orientation.

### Effect of magnetic field and probe current magnitude

Other potential origins of the asymmetric transitions are the measurement current and dipolar magnetic fields. Because of the large difference in resistivity between AF and FM phases the measurement current could lead to correlations of phases due to local differences in Joule heating. However, we varied the measurement current by a factor of 5 (the power by 25) and did not see any significant change in the behaviour (see Supplementary Fig. 5).

**Figure 3:**
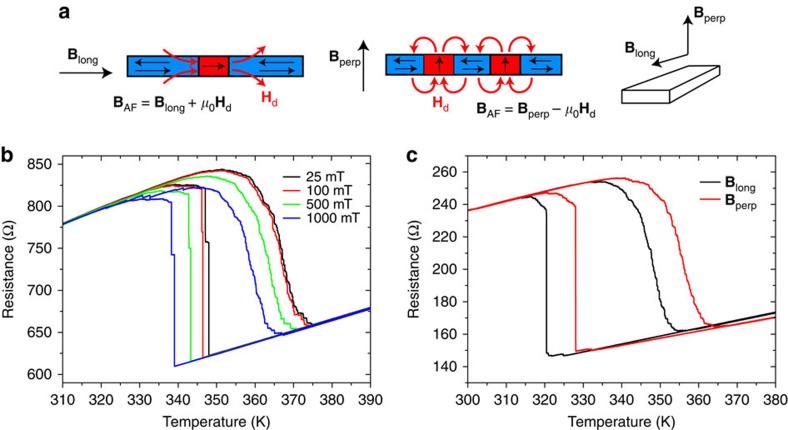
Effect of the magnitude and direction of the applied field. (**a**) Applied magnetic field aligns the FM phase in the directions longitudinal (**B**_long_) and perpendicular (**B**_perp_) to the stripe modifying the local effective fields acting on the AF phase due to different demagnetizing factors in the respective directions. (**b**) Longitudinal magnetic field induces an offset of the thermal hysteresis loop of about 9 K T^−1^, but no effect on the transition asymmetry is observed. (**c**) Effect of magnetic field of 1 T applied in the longitudinal and perpendicular directions. In the direction perpendicular to the stripe the demagnetizing field almost entirely compensates the applied field (the offset is 7.6 K). The data shown in **b** are for a stripe 220-nm-wide and 50-nm-thick, **c** a stripe 550-nm-wide and 33-nm-thick.

The dipolar magnetic fields arising from heterogeneous FM–AF phases are known to contribute to the width of the phase transitions^21^. The FM regions generate local dipolar fields on the order of hundreds of mT that together with an external magnetic field could alter the transition temperature of neighboring AF regions (as shown schematically in Fig. 3a). The phase transition temperature shift is roughly −9 K per 1 T of external magnetic field. Thus, the fields generated by the FM phases can potentially perturb the transition of neighboring AF regions. For an in-plane external magnetic field, the dipolar fields generated by a FM region in a film can either add or subtract to the external field depending on whether the FM and AF regions are collinear or orthogonal to the external field. However, for the stripes with the external field aligned with the stripe axis the dipolar fields from the FM regions will mostly add to the external field, potentially triggering the phase transition of neighboring regions (Fig. 3a). Conversely, for out-of-plane external magnetic fields the dipolar fields from the FM region will oppose the external field and hinder the nucleation of the FM phase in neighboring AF regions. Similar arguments can be used to examine the internal demagnetizing fields within the FM grains during cooling. These effective fields could lead to correlated behaviour that is qualitatively different for AF-to-FM and FM-to-AF transitions.

To test the role of dipolar fields we measure the response of a patterned stripe with magnetic fields applied either parallel to the stripe (longitudinal field) or perpendicular to the stripe and the sample plane (see Fig. 3b,c). In case of the longitudinal geometry we also varied the field magnitude (Fig. 3b). We observe a mean-field shift of the average transition temperatures dependent on the magnitude of the applied field, as well as on the different directions of the applied field. However, the stripes show the same transition asymmetry even though the contribution of the dipolar fields from the FM regions acting on the AF regions has the opposite sign for longitudinal and perpendicular applied fields.

## Discussion

After eliminating other potential sources we argue that the colossal asymmetry and reduced phase separation upon cooling observed on the mesoscale arises from the qualitative differences in the magnetic correlations in the FM regions compared with the AF regions. FM correlations are generally robust to local disorder^51^, whereas most studies of AF systems show the magnetic correlations are shorter-range^52^,^53^,^54^,^55^. It is well known that long-range FM order persists even in granular or amorphous films^51^. In contrast, AF order is found to be sensitive to local structural disorder such as stacking faults or grain boundaries where the AF correlation lengths are limited by the crystalline correlations length. For instance, thin granular AF films such as those used for exchange biasing in magnetic recording heads exhibit a blocking temperature that results from thermal fluctuations of individual AF grains^52^. The behaviour of these granular AF films is successfully modelled considering only the magnetic energy of individual grains and ignoring any magnetic correlations between AF grains. Even in high-quality epitaxial AF films the magnetic correlations, as measured by neutron scattering, are observed to be shorter or equal to the structural order^53^,^54^. Similarly, it is known from exchange bias studies using FeRh films that there is weak exchange coupling between the FM and AF phases compared with direct FM exchange^55^. The limited AF correlations is also reflected in the laser-induced AF–FM phase transition of FeRh where the exponential growth of ferromagnetic phase with time after excitation was explained by a model where nucleation of independent FM domains is dominant while the growth of existing domains is suppressed^42^. The small resistivity steps we observe upon warming indicate that individual AF regions (of a typical area of 0.005 μm^2^) undergo a first-order transition from AF to FM phases. This confirms that there is little correlation between AF regions^48^, which makes the AF-to-FM transition in the FeRh stripes very similar to that observed for the full films. This is in line with the assumption that the short-range AF correlations should not change with mesoscale patterning.

Besides the short AF correlations, residual FM phase in FeRh films at low temperatures represents another cause for the lack of superheating^49^, as it forms nucleation sites for the AF–FM transition. The residual FM phase is generally attributed to strain relaxation and has been previously observed at the interface with the substrate^43^, capping layer^56^ and even in the first few monolayers of a free-standing surface^57^. The work by Bennett *et al*.^58^ shows the full depth distribution of the low-temperature FM phase, indicating it is confined close to the film surfaces and does not break up the structure into many phase-separated domains^59^. In spite of these observations we do not find avalanches through the remaining AF phase and the AF–FM transition is not triggered before reaching the transition temperature of the remaining AF clusters.

However, in the case of the FM-to-AF transition the parent FM phase is homogeneous and it is stabilized by robust ferromagnetic interactions. Furthermore, according to our findings the patterned stripes show significantly larger supercooling compared with that observed in the full film^49^. When an AF region finally nucleates in the stripe the symmetry of ferromagnetic exchange interaction is broken and the AF phase will propagate through the stripe resulting in a sharp transition^60^. This behaviour is not observed in the full films since there are always nucleation sites (most likely a low density of non-magnetic inclusions in the films or residual AF clusters) to initiate the FM-to-AF transition. In the case of mesoscale stripes patterned below the typical nucleation site separation, the probability of finding one of these nucleation sites within the patterned area is reduced.

**Figure 4:**
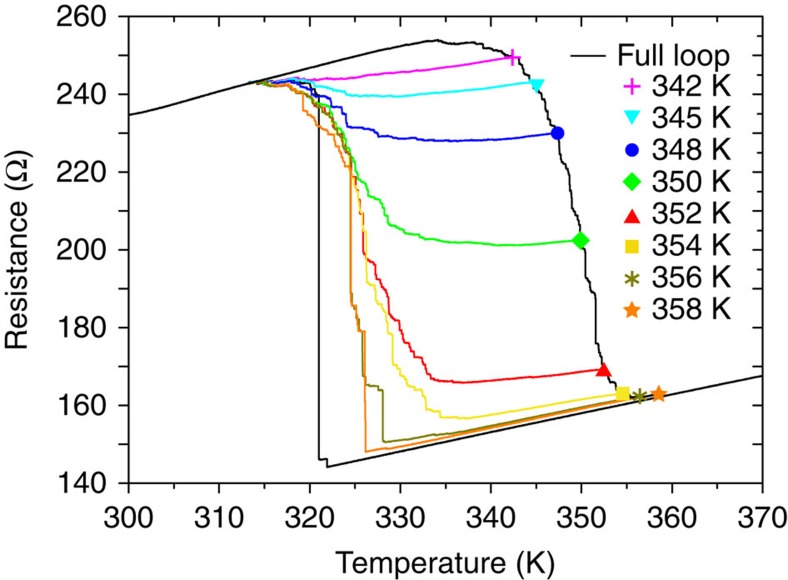
Temperature-driven hysteresis loops for an FeRh stripe. Transport measurements of a 33-nm-thick, 550-nm-wide and 4-μm-long stripe. The black curve represents a major loop showing the full temperature hysteresis and the asymmetry of the AF–FM and FM–AM transition. Colour curves show minor loops. For each minor loop the sample is heated only to the temperature shown by the symbols before the AF-to-FM transition is complete. The sample is subsequently cooled to the same temperature as the major loop. This way the content of residual AF domains upon cooling is controlled and the minor loop curves show gradually reduced supercooling and multiple-step transition as the remaining AF clusters break the FM correlation.

We can test this hypothesis by measuring minor hysteresis loops such as was done in ferromagnetic films^61^. In Fig. 4 we compare the resistance versus temperature loops in a single FeRh stripe where the AF-to-FM phase transition is not completed to the full thermal hysteresis. Stopping before a complete transition from the AF phase into the FM phase leaves residual AF domains in the stripe. Even stopping at 358 K where the AF-to-FM phase transition is 99.5% complete (as estimated by the change in resistance) results in a dramatic change of the FM-to-AF transition, which occurs at a much higher temperature and via multiple jumps. Warming to 354 K where the transition AF-to-FM transition is 97% complete results in the FM-to-AF transition occurring in many resistance steps and is qualitatively similar to that observed for a full film. This shows that only a few AF domains are enough to break the ferromagnetic exchange, nucleate the transition and dramatically change the shape of the FM–AF transition. Notably, initiating minor loops on the cooling curve (see Supplementary Fig. 4), leaving residual FM domains in the AF phase before warming, does not significantly affect the nature of the AF-to-FM transition which is consistent with the limited exchange between neighboring AF domains and AF–FM domains^55^ and lower magnetic correlation in the AF phase^48^. Furthermore, we see qualitatively similar behaviour when cycling through the transition by magnetic fields at a fixed temperature (see Supplementary Fig. 6). This clearly indicates the asymmetry itself is not due to thermally activated processes and the phase-separated state is reproducible upon repeated temperature or magnetic field sweeps.

In contrast, the step-like transition observed in manganite stripes^32^ shows randomness in the formation of the phase-separated state. The conductive pathway is different in each temperature cycle and a large jump in the resistance is observed when a manganite grain undergoes MIT, that is, it is not a result of correlated behaviour.

The changes to the FM-to-AF phase transition with patterning is analogous to what has been observed for magnetic reversal of ferromagnetic films^61^. Full films exhibit much smaller coercive fields than expected from the magnetic moment and anisotropy. This general behaviour was historically known as Brown's paradox^62^. It is now well understood that this low coercive field arises because magnetization reversal occurs via nucleation of reverse domains at predetermined nucleation sites in the films followed by domain-wall propagation. In homogeneous films, the magnetic fields needed to move a domain wall is much lower than the fields required to nucleate reversal in the absence of a defect. Patterning the film into mesoscale islands such that there are no longer any nucleation sites that initiate reversal within the area of the patterned region significantly enhances the coercive field. The magnetic reversal in single islands then proceeds via a single Barkhausen jump when a field-driven nucleation occurs^63^. The switching behaviour of single islands is qualitatively different from the extended film even when the island size is large compared with the ferromagnetic domain size and exchange length.

In conclusion, we have uncovered an unexpectedly large asymmetry of the first-order AF-to-FM metamagnetic phase transition between warming and cooling in mesoscale FeRh stripes. While modest asymmetries have been observed in the studies of FeRh films^21^,^48^,^49^, these effects are enhanced by orders of magnitude at the mesoscale where the difference in magnetic correlations of the AF and FM phases may lead to an almost complete suppression of the phase separated state upon cooling. We believe these results should be a general behaviour of first-order metamagnetic phase transitions of materials confined to the nano- and mesoscale. The nature of phase transitions initiated in AF or nonmagnetic phases should be much less sensitive to lateral confinement than the first-order transitions that initiate in the FM phase. This results from the robustness of the FM exchange to local strain and disorder when compared with the AF exchange. These results will have to be considered for devices and applications utilizing FeRh^23^,^39^,^40^ or related materials and may be potentially exploited in complex phase-transition materials, provided a transition specific order parameter can be effectively stabilized and controlled.

## Methods

### Film growth and lithography

Epitaxial FeRh films were grown on MgO (001) substrates at 450 °C and an argon pressure of 1.5 mTorr by d.c. magnetron sputtering using an equiatomic target. The films were post-annealed at 800 °C for 45 min and subsequently coated with a 2-nm Pt layer. The crystallographic orientation is such that [100] direction of FeRh aligns with [110] direction of MgO (see Supplementary Fig. 1). The FeRh films were patterned into stripes by e-beam lithography and ion-beam etching. The Au/Ti transport leads were subsequently made by ultraviolet lithography combined with the lift-off technique.

### Structural characterization using X-ray diffraction

Supplementary Figures 1–3 contain X-ray diffraction data for a typical 45-nm-thick FeRh (001) film on MgO (001) employed for this study. Symmetric and asymmetric (*hkl*) reflection data at room temperature are shown in Supplementary Fig. 1 for MgO and FeRh. Here the average FeRh crystallite size in the out-of-plane direction is retrieved via the Williamson and Hall analysis of the (00*l*)-peaks^64^. Furthermore, we followed the analysis by De Vries *et al*.^65^ to quantify the substitutional disorder in terms of the chemical order parameter *S*=*r*_α_−*r*_β_–1, with *r*_α_ and *r*_β_ being the fraction of Fe and Rh atoms at their corresponding sites, respectively. *S* can be experimentally determined from X-ray diffraction *θ*−2*θ* scans as^65^


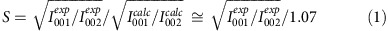


where 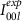 and 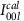 are the measured and theoretical (*00l*)-peak integral intensities, respectively.

### Electrical transport measurement

The electrical transport measurements were performed using a Keithley SourceMeter 2400 in both the 4-probe and 2-probe configurations. However, as no qualitative difference in the resistance of the FeRh stripes upon temperature sweeping was observed, we used the 2-probe configuration to increase the number of measured samples. The probe current was 30 μA. The temperature was swept at a rate of 1 K min^−1^ and a single resistance measurement was taken every 0.5 s, that is, the data are separated by a temperature step of 0.008 K. Unless stated otherwise, all measurements were performed in an applied field of 1 T.

### Data availability

The data that support the findings of this study are available from the corresponding author upon request (vojtech.uhlir@ceitec.vutbr.cz).

## Additional information

**How to cite this article:** Uhlíř, V. *et al*. Colossal magnetic phase transition asymmetry in mesoscale FeRh stripes. *Nat. Commun.* **7,** 13113 doi: 10.1038/ncomms13113 (2016).

## Supplementary Material

Supplementary Information Supplementary Figures 1-6
