# Supplementary material for: Colossal magnetic phase transition asymmetry in mesoscale FeRh stripes
Source: Nat Commun. 2016 Oct 11;7:13113. doi: 10.1038/ncomms13113 (PMC5062592; doi:10.1038/ncomms13113)
Supplement: Supplementary Information — Supplementary Figures 1-6 [file ncomms13113-s1.pdf]

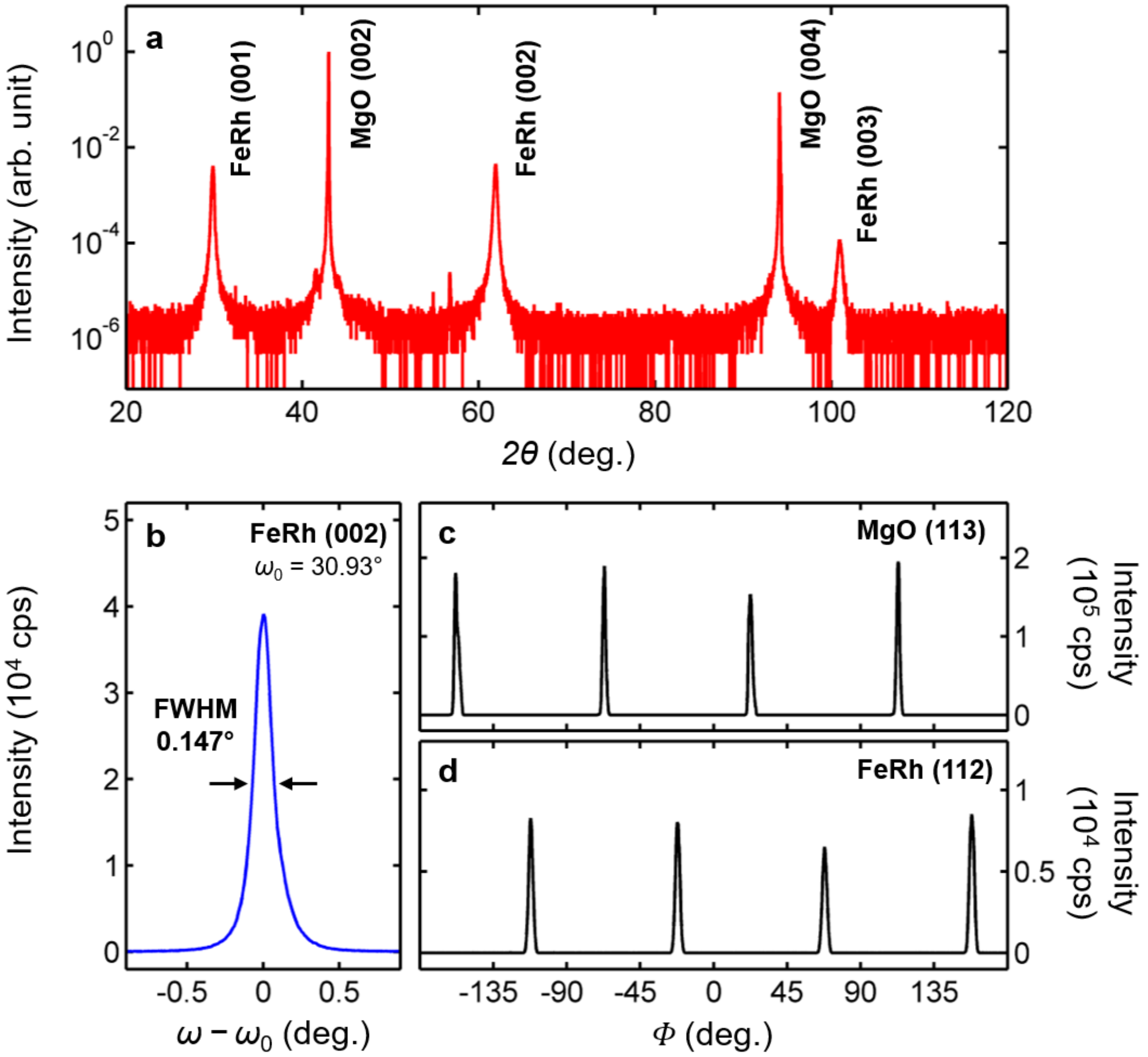

Supplementary Figure 1 | θ-2θ x-ray diffraction scan of an FeRh film on MgO (001) substrate. a, X-ray diffraction scan for a 45-nm thick FeRh (001) film grown epitaxially onto a MgO (001) substrate. The line-profile analysis of 2θ scans resulted into an average crystallite size of 45 ± 2 nm and the chemical order parameter S = 0.78. **b,** An ω scan about the FeRh (002) peak yields a mosaic spread full width at half maximum of 0.147 degrees. **c**, **d**, Azimuthal x-ray diffraction scans, where the MgO (113) and FeRh (112) diffraction peak intensities are monitored as the sample is rotated. The four-fold symmetry and the 45 degree shift between the peaks in **c** and **d** confirm the epitaxial in-plane orientation with the FeRh [100] being parallel to the MgO [110].

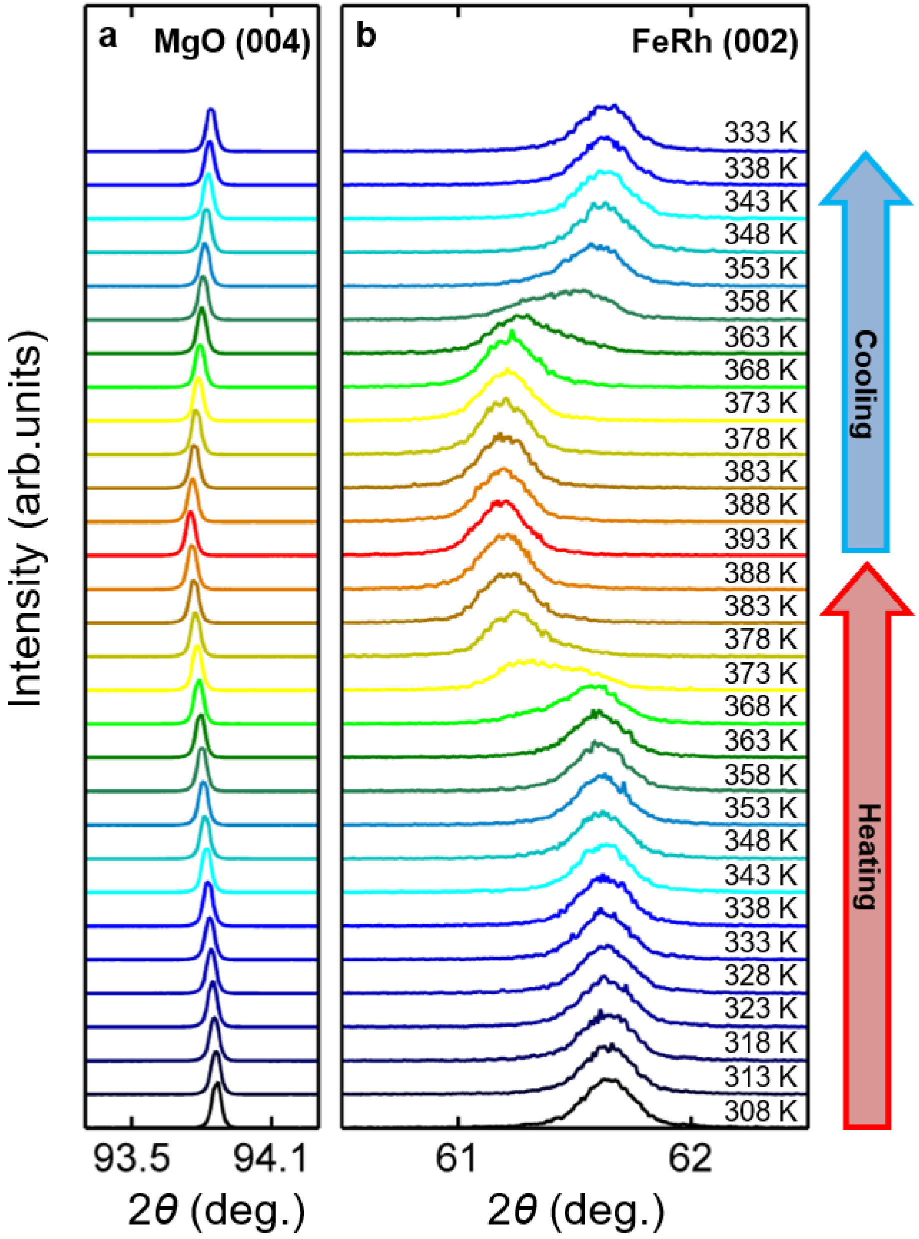

Supplementary Figure 2 | Temperature-dependent x-ray diffraction. a, **b,** Temperature-dependent θ-2θ x-ray diffraction scans near angular regions of the MgO (004) and FeRh (002) peaks, respectively, measured for a 45-nm-thick FeRh (001) film grown epitaxially onto a MgO (001) substrate. The x-ray scans have been offset for clarity and scans were taken sequentially starting at 308 K, heating to 393 K and then cooling to 333 K.

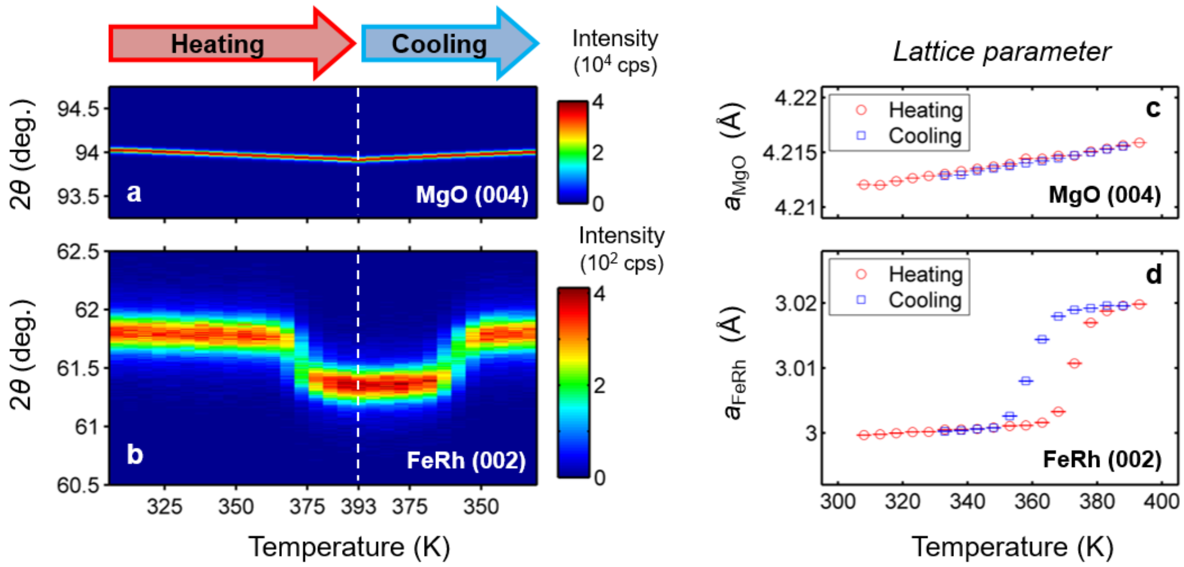

Supplementary Figure 3 | Temperature-dependent x-ray diffraction. a, **b**, Temperature-dependent θ-2θ x-ray diffraction scans for a 45-nm-thick FeRh (001) film grown epitaxially onto a MgO (001) substrate, in the angular regions near the MgO (004) and FeRh (002) peaks. The results are derived from the scans shown in Supplementary Fig. 2 which are replotted as color-coded intensity maps. **c**, **d**, Average lattice constant vs. temperature for the MgO substrate and the FeRh films, showing the hysteresis in the FeRh phase transition. The lattice parameter values and the error bars are derived from the Gaussian line profile analysis of the MgO (004) and FeRh (002) peaks via least squares fitting.

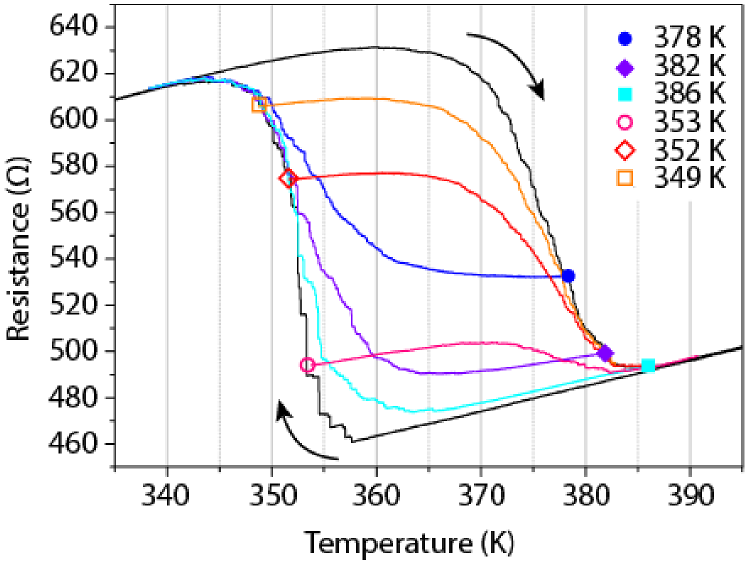

Supplementary Figure 4 | Resistance vs. temperature of an FeRh stripe on Al_2O_3 substrate. Resistance vs. temperature of a 30-nm-thick, 400-nm-wide and 3.8-μm-long stripe. Multiple steps in the transition upon cooling indicate increased structural disorder due to (111) growth. The minor loops initiated upon heating (full symbols) and cooling (open symbols) are plotted. The minor loops initiated upon cooling do not show any change of the transition character upon warming with respect to the major loop. The minor loops initiated upon heating just before the completion of the AF-FM transition show a clear difference of the transition step size upon cooling with respect to the major loop – the long-distance FM correlation is broken by the AF residuals.

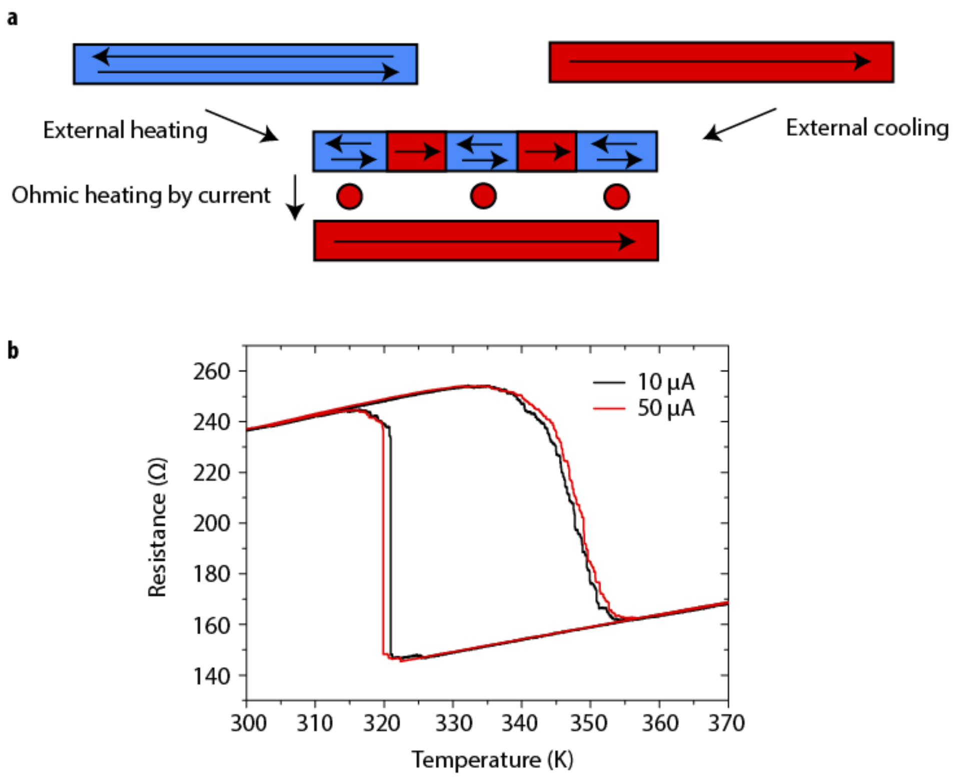

Supplementary Figure 5: | Effect of probe current. a, The schematic FeRh stripe is driven by temperature or magnetic field from either AF or FM phase into a phase-separated state. Once there, the probe current preferentially heats the AF phase due its higher resistance and thus stabilizes the FM phase. **b,** This effect did not influence the transition asymmetry while the probe current was varied by a factor of 5.

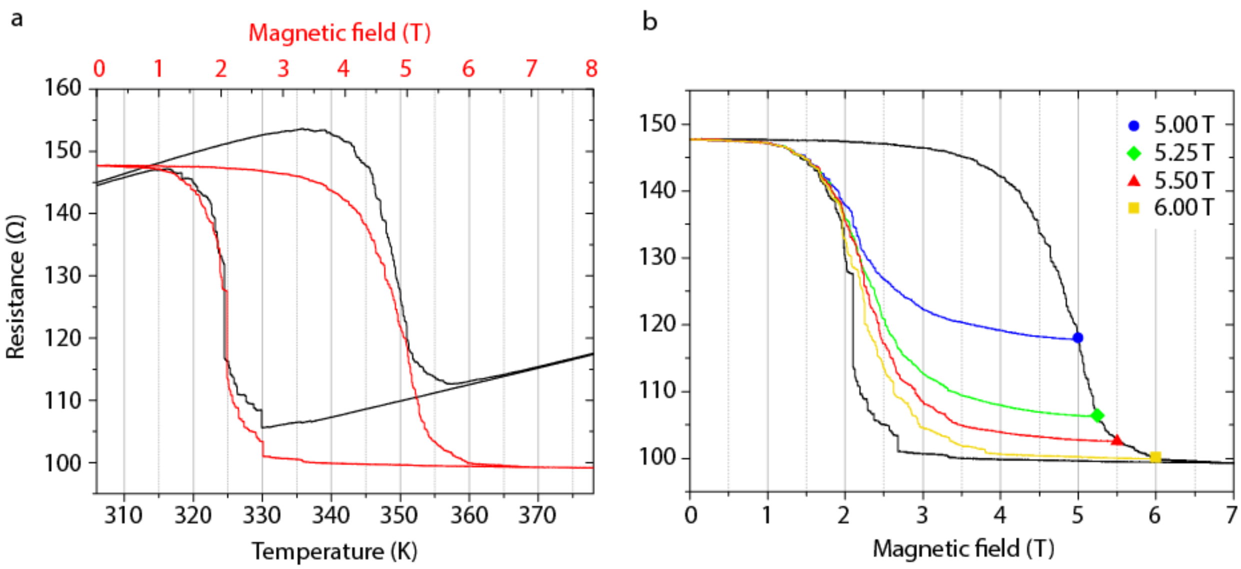

Supplementary Figure 6 | Field-driven magnetic phase transition. a, Correspondence of the magnetic field- and temperature-driven phase transition hysteresis for a 33-nm-thick, 1.3-µm-wide and 4-µm-long stripe. The field hysteresis is taken at a temperature of 315 K, the temperature hysteresis at a field of 1 T. **b,** Minor loops initiated prior to completion of the AF-FM transition show qualitatively similar behavior as the temperature-driven ones: the AF residuals break the FM correlation and the transition becomes continuous.
